# Invasive Streptococcus pyogenes infection: a case report

**DOI:** 10.1099/acmi.0.000767.v3

**Published:** 2024-07-08

**Authors:** Samia Bazhar, Yassine ElBenaissi, Elmostafa Benaissa, Yassine Ben Lahlou, Mariama Chadli

**Affiliations:** 1 Department of Clinical Bacteriology, Mohammed V Military Teaching Hospital, Faculty of Medicine and Pharmacy, Mohammed V University, Rabat, Morocco; 2 Research Team of Epidemiology and Bacterial Resistance, Faculty of Medicine and Pharmacy, Mohammed V University, Rabat, Morocco; 3 Center for Virology, Infectious and Tropical Diseases, Mohammed V Military Teaching Hospital, Rabat, Morocco

**Keywords:** invasive infection, *Streptococcus pyogenes*, septic arthritis

## Abstract

The Group A Streptococcus (GAS), also known as *Streptococcus pyogenes* (*S. pyogenes*), is a human pathogen causing various infections, ranging from mild, such as tonsillitis and impetigo, to severe and invasive conditions like septicemia and necrotizing fasciitis. Despite a decline in incidence and severity during the twentieth century due to antibiotics, there has been a reported increase in severe cases since the 1980s in industrialized countries. *S. pyogenes* is a human pathogen with a natural reservoir in the pharynx and skin, exhibits asymptomatic carriage in various body sites. It is responsible for a spectrum of clinical manifestations, from asymptomatic carriage to severe invasive infections. Transmission occurs through respiratory droplets or direct contact with skin lesions. Bacteriologically, *S. pyogenes* is a Gram-positive β-hemolytic streptococcus. This summary highlights a case of invasive Group A Streptococcus infection in a 28-year-old diagnosed at the microbiology laboratory of the Mohammed V Military Training Hospital in Rabat, Morocco. A 28-year-old patient, without any specific medical history, presented with acute febrile oligoarthritis. Following a recent flu-like syndrome and febrile tonsillitis, the patient experienced asymmetric inflammatory oligoarthralgia affecting the left knee, left ankle, and right shoulder, accompanied by functional impairment of the left lower limb. Upon admission, clinical examination revealed swelling, positive patellar tap, and sternal involvement. Laboratory and imaging findings indicated an abscessed collection in the left knee and anterior mediastinitis. Emergency aspirations revealed Group A Streptococcus, specifically *Streptococcus pyogenes*, leading to a diagnosis of septic arthritis. Dual antibiotic therapy and knee joint drainage resulted in symptom resolution after 45 days. The rise in severe Group A Streptococcus infection underscores the need for early detection and treatment. Widely sharing the French High Council for Public Health’s antibiotic prophylaxis recommendations is crucial for awareness. Collaborating between clinicians and microbiologists is essential for effective management.

## Data summary

No data was generated during this research or is required for the work to be reproduced.

## Introduction

The Group A Streptococcus (GAS), or *Streptococcus pyogenes* (*S. pyogenes*), is a strictly human pathogen capable of causing a wide range of infections, either benign such as tonsillitis [1] and impetigo, or severe and invasive such as septicemia, streptococcal toxic shock syndrome (STSS), necrotizing fasciitis and septic arthritis [2]. Pyogenic septic arthritis is a serious infection associated with the risk of joint destruction and infection dissemination, impacting both functional and vital prognosis [3].

In the course of the twentieth century, these infections experienced a significant decrease in both their incidence and severity, primarily due to the advent of antibiotic therapy. However, an increase in the frequency of severe infections, sometimes in the form of clustered cases, has been reported since the early 1980s in several industrialized countries [4].

Globally, invasive *S. pyogenes* infections are estimated at 663000 new cases and 163000 deaths annually. Comparable incidences ranging from 1.5 to 5.2 cases per 1000000 inhabitants are reported in European and North American countries. The mortality associated with these invasive *S. pyogenes* infections is estimated between 12.5 and 19 %, rising to 45 % when STSS complicates the clinical form [5].

We present a case of invasive Group A Streptococcus infection in a 28-year-old adult diagnosed at the microbiology laboratory of the Mohammed V Military Training Hospital in Rabat Morocco.

## Case presentation

This concerns a 28-year-old patient admitted to the emergency department for acute febrile oligoarthritis evolving for 7 days, without any specific medical history. One month before hospitalization, he had presented with a flu-like syndrome associated with febrile tonsillitis, the patient was prescribed a five day course of paracetamol, vitamin C and a cough suppressant, with good clinical improvement at the end of treatment.

Twenty-one days later, the patient reported the rapid onset of asymmetric inflammatory oligoarthralgia involving the left knee, left ankle, and right shoulder, leading to total functional impairment of the left lower limb, associated with swelling of the sternocostal region. This evolution occurred in the context of a general state alteration, unquantified fever, anorexia, and unquantified weight loss outside of any medication.

Upon admission, the patient was stable neurologically, hemodynamically, and respiratorily, with a Glasgow’s score (GCS) of 15, normotensive at 11/7, tachycardic at 108 beats min^−1^, polypneic at 22 cycles min^−1^, SaO_2_ at 98 %, and a central temperature at 38 °C. Conjunctivas were normally coloured, the throat was clean with poor oral hygiene.

The musculoskeletal examination showed painful swelling of the left ankle and left knee with filling of the subquadricipital recess and a positive patellar tap on palpation. The Womac score was 88. The hips were free and painless, but there was a painful satellite adenopathy in the left inguinal region.

**Fig. 1.**
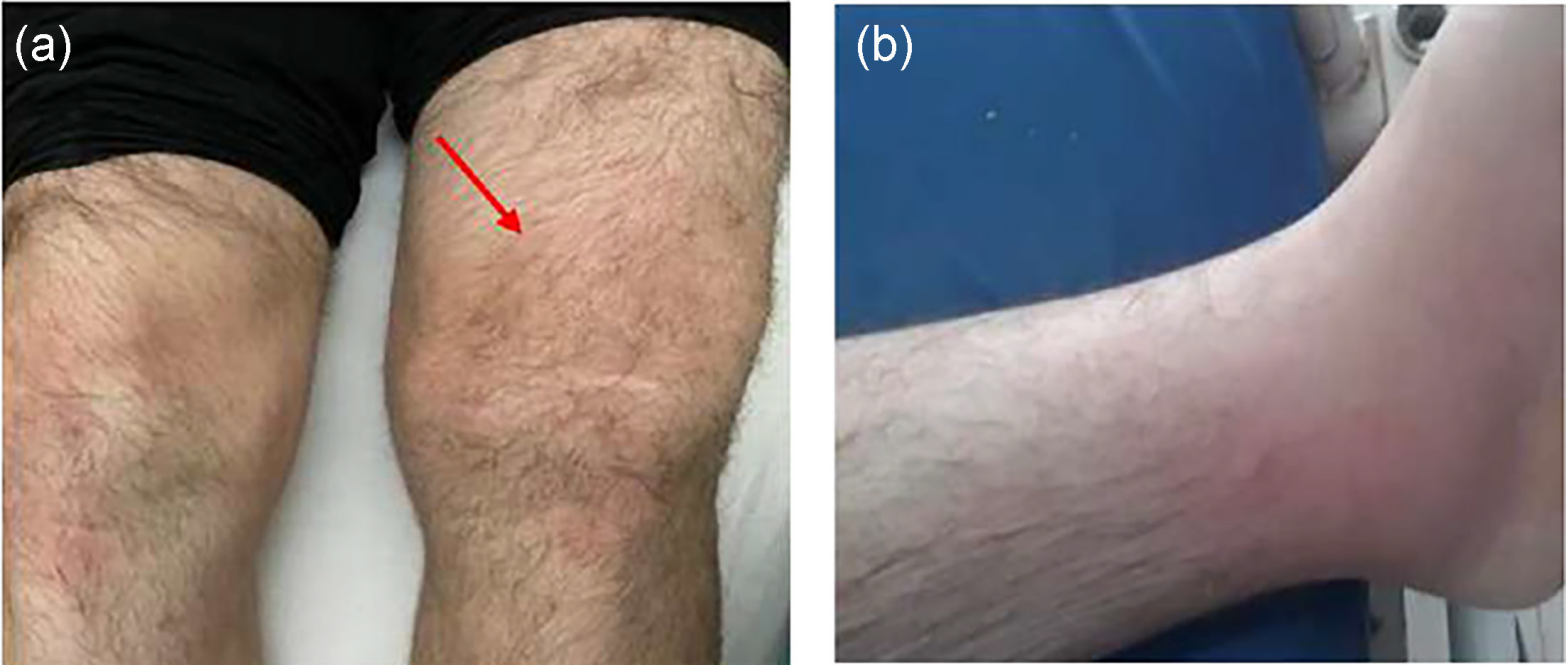
Painful swelling of the left ankle and left knee.

In the thorax, there was a painful anterior sternal swelling, irregular in contour, soft and mobile in the deep plane. The skin examination revealed an erythematous, edematous patch on the anterior surface of both ankles and legs with infiltration of the soft tissues of the left leg (Fig. 1). Additionally, there were no wounds, fistulas, or pustules.

The rest of the clinical examination was unremarkable. The initial biological assessment showed leukocytosis at 19 500 cells mm^−^³, with 89.4 % neutrophils and lymphopenia at 1 300 cells mm^−^³. There was a clear inflammatory syndrome with a serum C-reactive protein (CRP) level of 453 mg l^−1^, ferritin above 1 600 ng ml^−1^, corrected calcium at 104 mg l^−1^, uric acid at 24 g l^−1^. Renal function was normal, proteinuria was 435 mg l^−1^, and the hepatic assessment was unremarkable. HIV, HCV, and HBV serologies were negative.

The infectious assessment noted negative aerobic-anaerobic blood cultures and a sterile urine culture.

**Fig. 2.**
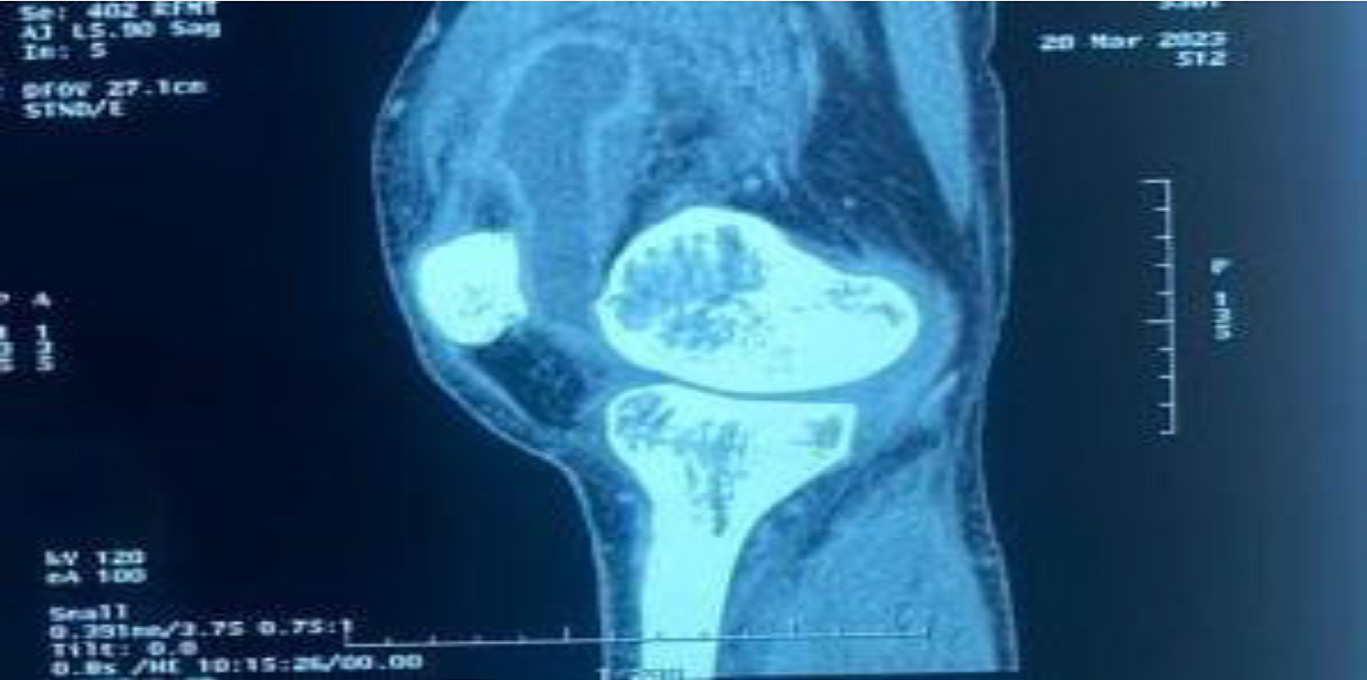
MRI of the left knee showing an abscessed collection extending from the subquadricipital recess to the lateral external recess, measuring 22×99 mm, with hypodensity.

The Magnetic Resonance Imaging (MRI) of the left knee revealed an abscessed collection extending from the subquadricipital recess to the lateral external recess, measuring 22×99 mm and showing hypodensity, associated with infiltration of adjacent soft tissues (Fig. 2).

**Fig. 3.**
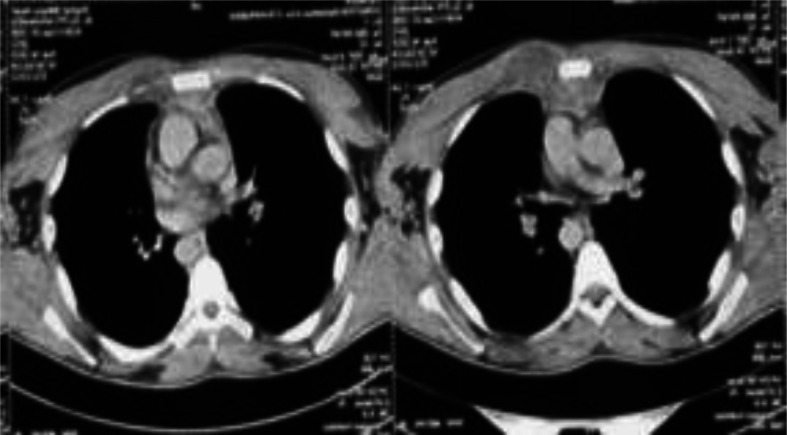
Thoracic CT scan showing a pre- and retrosternal collection extending towards the cervicothoracic orifice, measuring 63×74×97 mm, with sternal erosion and the onset of anterior mediastinitis.

The thoraco-abdomino-pelvic computed tomography showed a pre- and retrosternal collection extending towards the cervicothoracic orifice, measuring 63×74×97 mm, with sternal erosion and the onset of anterior mediastinitis (Fig. 3).

**Fig. 4.**
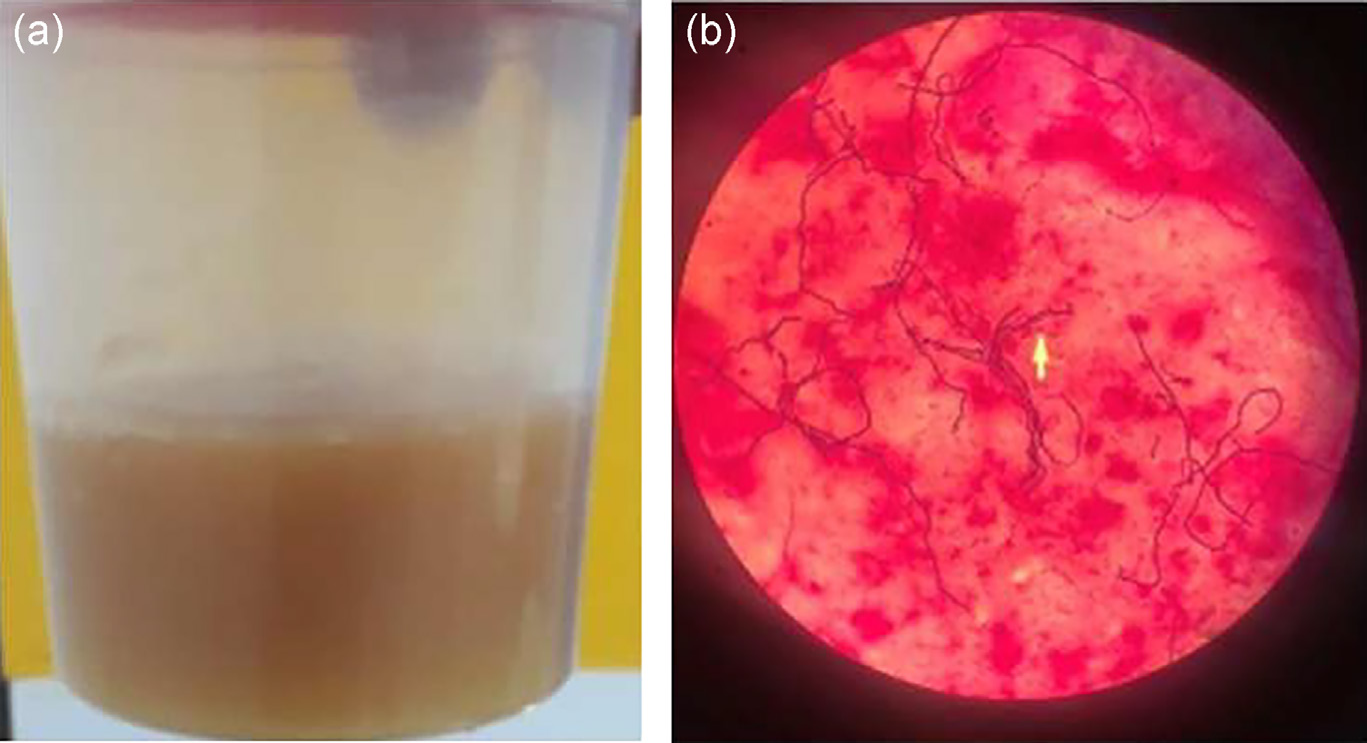
Microbiological examination of the joint aspiration. (a) Macroscopic appearance of joint fluid after puncture. A purulent and hemorrhagic fluid in a context of septic arthritis where major synovial inflammation is responsible for intra-articular bleeding. (b) *S. pyogenes* in cocci short chains, under Gram staining. Their blue color indicates that they are Gram positive. Optical microscope with magnification ×100.

Faced with this clinical presentation of left knee arthritis associated with a probable infectious anterior mediastinitis, emergency ultrasound-guided fine needle aspiration of the joints and mediastinum were performed, supplemented by a cyto-bacteriological examination of the samples, revealing a turbid aspect (Fig. 4). Cytology showed 28800 mm³ of lymphocytes, 4800 mm³ of erythrocytes, with a leukocytic formula consisting of 99 % polymorphonuclear neutrophils (PMNs). The samples have benefited from a microscopic examination after Gram-staining and showed a Gram-positive cocci in short chains. The cultures were carried out on Columbia agar with 5 % blood (GS), and Polyvitex chocolate agar (GSC). All these media were incubated at 37.8 °C for 18 to 24 h in atmospheres enriched with 5–10 % of CO_2_.

Aerobic-anaerobic culture yielded a strongly positive result for numerous Group A Streptococcus. The aerobic-anaerobic culture was positive, isolating and identifying *Streptococcus pyogenes*. The identification was based on morphological, Streptococcal grouping kit using latex agglutination, cultural and biochemical characters (gallery Api 20 Strep).

The antibiotic sensitivity study was determined using the agar diffusion method on Mueller-Hinton agar (MH), following the recommendations prescribed by the European Society of Clinical Microbiology and infectious Diseases (CA-SFM) [6] (Table 1).

**Table 1.** The antibiotic sensitivity

Antibiotic	Group A Streptococcus	
	Critical diameter mm	
	S ≥	R <
Penicilline G	18	18
Gentamycine	17	–
Tetracycline	23	23
Erythromycine	21	18
Pristinamycine	22	22
Clindamycine	21	18
Linezolide	19	19
Vancomycine	13	13
Teicoplanine	15	15
Moxifloxacine	19	19

Thus, the diagnosis of septic arthritis due to Group A streptococcus (pyogenic) was established, and the patient received dual antibiotic therapy comprising ceftriaxone 2 grams per day and ciprofloxacin 500 mg twice daily. This was followed by knee joint drainage. The clinical and biological response to treatment was marked by the resolution of inflammatory symptoms after 45 days of antibiotic therapy.

## Discussion

*Streptococcus pyogens* is a human pathogen with a natural reservoir in the pharynx and skin. It can be isolated in asymptomatic carriers from the nasopharynx, skin, vagina, or rectum. The bacterium is responsible for a wide range of clinical manifestations, from asymptomatic carriage to severe invasive infections that can rapidly compromise the prognosis [5]; these include: tonsillitis, sore throat, scarlet fever, impetigo and septic arthritis. Our patient presented with the flu-like syndrome and febrile tonsillitis a month before hospitalization, this can be in favour with the septic arthritis that’s affected the patient later knowing that all these infections are caused by the same bacteria *S. pyogens*.

A case of septic arthritis caused by *S. pyogenes* infection was first reported in 1984, and in that case, a serious infection caused by *S. pyogenes* occurred in a nursing home patient and resulted in sepsis, necrotizing fasciitis, septic arthritis, and cellulitisRuben et al., 1984. Septic arthritis after toxic shock syndrome caused by *S. pyogenes* was reported in 1995González-Ruiz and Ridgway, 1995. *S. pyogenes* infection causing multifocal septic arthritis has also been reportedMarti and Anton, 2007. Previous research has shown that *S. pyogenes* can invade the joint microenvironment, and be a step in the development of septic arthritisLe Hello et al., 2009 Volzke et al., 2020inoculated mice intravenously with *S. pyogenes* and observed septic arthritis 3~20 days after infection with increased levels of interleukin (IL)-1β and IL-6 in the joints along with an increased amount of nuclear factor (NF)-κB receptor activator ligand, which is a key cytokine for osteoclast formation. Septic arthritis occurs in lower limb joints in 90 % of cases, with the hip being the most commonly involved, followed by the kneeWang et al., 2003;Xu et al., 2016. Sternoclavicular joint involvement has also been reportedDhekane et al., 2020;Kwon et al., 2020. Joint pain (81 %) is the most common presentation, followed by fever, swelling and limitation of movement, and 89 % of patients show an increased ESR (≥20 mm h^−1^) Wang et al., 2003[7].

Interhuman transmission of *S. pyogenes* occurs through droplets from the upper respiratory tract or direct contact with skin lesions. Bacteriologically, *S. pyogenes* is a Gram-positive β-hemolytic streptococcus, catalase-negative, and oxidase-negative. It is a facultative anaerobe, thrives better in 5–10 % carbon dioxide, and forms distinct colonies on blood agar plates.

Among the numerous virulence factors of *S. pyogenes*, the M protein holds a special place. It is a surface protein that constitutes a major virulence factor, and it plays a fundamental role in typing *S. pyogenes* strains [5].

Prevention strategies for community-acquired invasive GAS infection were established on 18 November 2005, by the French High Council of Public Hygiene (CSHPF) [8].

The prevention strategy for isolated or clustered cases relies primarily on defining a case of invasive GAS infection, distinguishing between certain, probable, or possible cases. Our observation can be defined as a certain case of invasive GAS infection since *S. pyogenes* was isolated from a usually sterile fluid (joint fluid collected upon patient admission). The uniqueness of our observation lies in establishing the diagnosis of invasive GAS infection after isolating and identifying the microorganism through culture.

The CSHPF has defined several risk factors for acquiring invasive GAS infection in adults (Table 2) [8].

**Table 2.** Risk Factors for Invasive Group A Streptococcal Infection In Adults [8]

Risk Factors for Invasive Group A Streptococcal Infection In Adults
Age >65 years
Progressive chickenpox
Extensive skin lesions, including burns
Intravenous drug use
Underlying medical conditions (diabetes, cancer, hematologic disorders, HIV infection, heart failure)
Significant oral corticosteroid use (prednisone 5 mg/kg/day >5 days (recent treatment) or prednisone 0.5 mg/kg/day for 30 days)

In our case, the patient had no risk factors and no identifiable infectious entry point despite thorough investigation. In France, the CSHPF recommends initial antibiotic prophylaxis with an orally administered second or third generation cephalosporin for a duration of 8 to 10 days [6]. In case of cephalosporin contraindication, the use of an oral macrolide is recommended (Azithromycin for 3 days or Clindamycin for 10 days). Lastly, in the presence of macrolide-resistant strains, penicillin prophylaxis for 10 days is suggested, combined with rifampicin during the last 4 days of treatment [8].

## Conclusion

The current resurgence of invasive infections withGAS, their severity, and the urgency of initiating specific treatment underscore the importance of early recognition of these infections. The recommendations for antibiotic prophylaxis from the French High Council for Public Health (CHSPF) in cases of invasive GAS infections should be widely known and disseminated.

Given the complexity of the implicated pathogens and the issue of resistance, the safest approach is to collaborate closely between clinicians and microbiologists to ensure the best possible patient care.

## Abbreviations

CSHPF: French High Council of Public Hygiene
GAS: group A streptococcus
MRI: magnetic resonance imaging
*S. pyogenes*: *Streptococcus pyogenes*
STSS: streptococcal toxic shock syndrome

## References

[1] Martin JM (2010). Pharyngitis and streptococcal throat infections. Pediatr Ann.

[2] Caillet-Gossot S, Rousset-Rouviere C, Arlaud K, Dubus J-C, Bosdure E (2011). Clustered cases of intrafamily invasive Streptococcus pyogenes infection (or group A streptococcus). Arch Pediatr.

[3] Mhamdi G (2020). Pyogenic septic arthritis: clinical and Microbiological profile.

[4] Veyssier-Belot C, Lèvy-Bruhl D, Carret G, Bouvet A, Portier H (2004). Médecine et Maladies Infectieuses.

[5] Denis F, Christian Martin M-C, Cattoir V (2016). 28.3 Famille Des Streptococcaceae et Des Enterococcaceae.

[6] EUCAST (2023). Comité de l’antibiogramme de La Société Française de Microbiologie Recommandations.

[7] Yu D, Gao W, Guo D, Lu Q, Chen Y (2023). Case report: septic arthritis in children caused by *Streptococcus pyogenes-*rational use of antibiotics. Front Cell Infect Microbiol.

[8] CSHPF (2005). Opinion of the higher council of public health of France on the conduct to be taken around one or more cases of community origin of invasive infections with Streptococcus pyogenes (or group A Streptococci). http://www.hcsp.fr.

